# Toxicity of Parasporin-4 and Health Effects of Pro-parasporin-4 Diet in Mice

**DOI:** 10.3390/toxins6072115

**Published:** 2014-07-16

**Authors:** Shiro Okumura, Hironori Koga, Kuniyo Inouye, Eiichi Mizuki

**Affiliations:** 1 Biotechnology and Food Research Institute, Fukuoka Industrial Technology Center, 1465-5 Aikawa, Kurume, Fukuoka 839-0861, Japan; E-Mail: emizuki@fitc.pref.fukuoka.jp; 2 Division of Gastroenterology, Department of Medicine, Kurume University School of Medicine, 67 Asahi-machi, Kurume, Fukuoka 830-0011, Japan; E-Mail: hirokoga@med.kurume-u.ac.jp; 3 Division of Food Science and Biotechnology, Graduate School of Agriculture, Kyoto University, Sakyo-ku, Kyoto 606-8502, Japan; E-Mail: inouye@kais.kyoto-u.ac.jp

**Keywords:** *Bacillus thuringiensis*, Bt toxin, parasporin, mouse, kidney

## Abstract

Parasporin-4 (PS4) is an aerolysin-type β-pore-forming toxin produced by *Bacillus thuringiensis* strain A1470. It exhibits specific cytotoxicity against human cancer cell lines; therefore, it is expected to be useful for the diagnosis and treatment of particular types of cancer cells. We examined the acute toxicity of PS4 on ICR mice. The LD_50_ value was 160 μg/kg by a subcutaneous route. Potassium, ammonium, magnesium ion, creatinine, and urea nitrogen decreased in urine by the injection of PS4. Simultaneously, creatinine and urea nitrogen in mice serum increased. These results imply that PS4 impairs kidney function in mice. PS4 is obtained from Pro-parasporin-4 (ProPS4) by processing, and ProPS4 is produced by recombinant *Escherichia coli* as the inclusion body. The inclusion body of ProPS4 can be solubilized in a weak acid solution and activated by pepsin, implying that it would be solubilized and activated in the stomach of mammals after oral administration. Thus, the influence of the oral administration of it by C57BL/6J mice was examined. Although ProPS4 was activated to PS4 in the mouse digestive tract, any serious health hazard was not observed and there was no significant difference in body weight change.

## 1. Introduction

Parasporin is a type of parasporal protein produced by *Bacillus thuringiensis* (Bt) and related bacteria that is capable of preferentially killing cancer cells [1]. Bt toxin is well known as an insecticidal protein produced in the parasporal inclusion that kills susceptible insects [2,3]. However, non-insecticidal Bt strains are more widely distributed than insecticidal ones [4], and we have discovered the strain producing a parasporin from many non-insecticidal strains [1]. At present, 19 parasporins have been designated into six first rank levels by the Committee of Parasporin Classification and Nomenclature [5]. Moreover, some parasporin-like proteins without registration have been reported all over the world [6,7,8].

Parasporin-4 (PS4; it is also designated as Cry45Aa by the *B. thuringiensis* δ-endotoxin nomenclature committee) is an aerolysin-type β-pore-forming toxin [9]. It exhibits strong cytotoxicity against several human tumor cell lines, especially CACO-2 cells [10]. Pro-parasporin-4 (ProPS4) is the precursor of PS4 and it is produced in the parasporal inclusion of strain A1470. ProPS4 is cleaved to form PS4 by protease treatment, which results in the cytocidal activity of particular cell lines. Since the strain also simultaneously produces parasporin-2 [11], ProPS4 produced by recombinant *Escherichia coli* as the inclusion body [12] was used in this study. It could be solubilized in acidic conditions and activated from the 31-kDa protoxin to the 27-kDa active form by pepsin cleavage of the C-terminus [13]. Moreover, the cytotoxic activity of the toxin was stable over a broad pH range (pH 2.0–11.0) [13], which imply that when ProPS4 is orally administered, it would be solubilized in gastric juice and activated by digestive enzymes in the stomach.

The digestive juice of most insects are alkaline [14], thus the parasporal inclusions of Bt toxins are generally solubilized in an alkaline buffer *in vitro*. Most Bt toxins including parasporins are considered to become inactive or in the form of insoluble aggregates in acidic conditions. However, because PS4 retains its cytotoxic activity in low pH conditions and it exhibits cytotoxicity to the CACO-2 cells derived from colon cancer, ProPS4 intake may be effective for the therapy of gastrointestinal cancer.

In this study, we examined the acute toxicity of PS4 on ICR mice in the scope for further application of PS4 for cancer therapy. We also examined activation and its health effects of orally administrated ProPS4 against C57BL/6J mice to explore the possibility for the treatment and prevention of gastrointestinal cancer.

## 2. Results and Discussion

### 2.1. Acute Toxicity of PS4 for ICR Mice

PS4 was injected into 7-week-old male ICR mice via subcutaneous injection with 100 μL phosphate buffered saline (PBS). Table 1 shows the dose amount and mortality at 48 h after administration. The calculated median lethal dose (LD_50_) per body weight was 0.16 mg/kg. Moreover, the 95% confidence limit of the value was 0.096–0.22 mg/kg.

Previous studies have reported the LD_50_ value of other bacterial pore-forming toxins for mice. For example, Streptolysin O from *Streptococcus pyogenes* was 8 μg/kg [15]; Listeriolysin O from *Listeria monocytogenes* was 3–12 μg/kg [15]; ε-toxin from *Clostridium perfringens* was 100 ng/kg [15]; and α-hemolysin from *Staphylococcus aureus* was 0.68 μg/kg [16]. Compared with these other toxins, PS4 has low toxicity for mice, which would be expected because health hazards of the Bt strain in nature have never been reported although the abovementioned bacteria are pathogenic. However, LD_50_ of 0.16 mg/kg cannot be considered to be low toxicity as an absolute value. Thus the hazard presented by PS4 is going to require further examination in detail.

**Table 1:** The dose amount of parasporin-4 (PS4) and mortality at 48 h after administration.

Dose amount (μg)	Number of administrations	Number of deaths	Mortality (%)
3	5	0	0
6	9	7	78
12	9	8	89
24	5	5	100
48	4	4	100

### 2.2. Activation of ProPS4 in the Digestive Tract of Mouse

**Figure 1:**
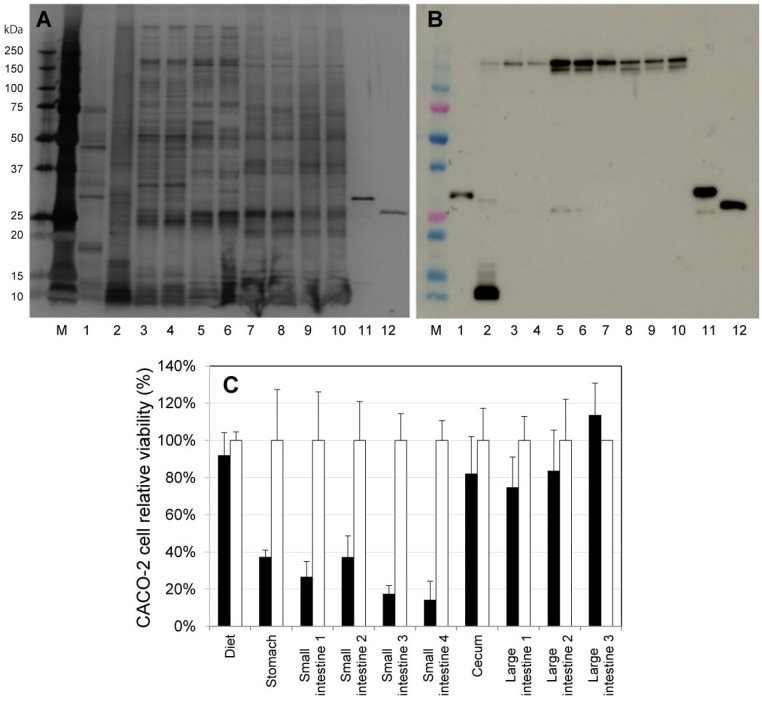
Activation of ProPS4 in the digestive tract of mouse. The contents in the small intestine were numbered from stomach side to cecum side. The contents in the large intestine were collected every fecaloma and numbered from cecum to anus. They were disrupted in the presence of glass beads (φ1.0 mm) using a bead cell disrupter and filtered using a centrifugal filter device (pore size, 0.45 μm). SDS*-*PAGE (**A**) and immunoblotting (**B**) were then performed. Lane M indicates the molecular size marker; Each lane indicates the diet (lane 1), content in the stomach (lane 2), in the small intestine 1–4 (lanes 3–6), in the cecum (lane 7), in the large intestine 1–3 (lanes 8–10), pro-parasporin-4 (lane 11), and parasporin-4 (lane 12). The filtered sample was added to a well of CACO-2 cells in a 96-well microplate, and cell viability was measured by the MTT assay at 20 h after administration (**C**). Black bars indicate the CACO-2 cell relative viability of samples with ProPS4, and white bars indicate those without ProPS4.

In this experiment inclusion body of ProPS4 produced by recombinant *E. coli* was used. Generally, high-level expression of recombinant protein in *E. coli* often results in the formation of inclusion body [17]. It is considered to be a result of misfolding or partially folded polypeptide [18]. Therefore, most recombinant proteins from inclusion bodies require a refolding step for their activity. However, PS4 prepared from recombinant ProPS4 reveals its own activity without refolding step [13]. Thus C57BL/6J mice were fed diets containing 10% inclusion body of ProPS4 under free-feeding conditions overnight. The contents in the digestive tract were then collected, and immunoblotting with anti-PS4 serum was performed (Figure 1A,B)*.* In the immunoblotting results, a band of ProPS4 was naturally observed in the lane of the diet sample (Figure 1B, lane 1). In the lane of stomach content, two minor bands of PS4 and PS4 multimer and a major band of degradation products of PS4 were observed (Figure 1B, lane 2). The bands of PS4 multimer were also observed in the lanes of the small intestine, cecum, and large intestine (Figure 1B, lanes 3–10). Moreover, the minor band of the PS4 monomer was observed in the lanes of small intestine 3 and 4 (Figure 1B, lanes 5 and 6). CACO-2 cell relative viability compared with the sample from contents without ProPS4 is shown in Figure 1C. The contents in stomach and small intestine 1–4 were significantly cytotoxic compared with the control. These results demonstrate that ProPS4 is solubilized and activated in the digestive tract of the mouse, and cytotoxic activity of the PS4 was retained in the stomach and small intestine.

### 2.3. Influence on Mouse Health of the Oral Administration of ProPS4

**Figure 2:**
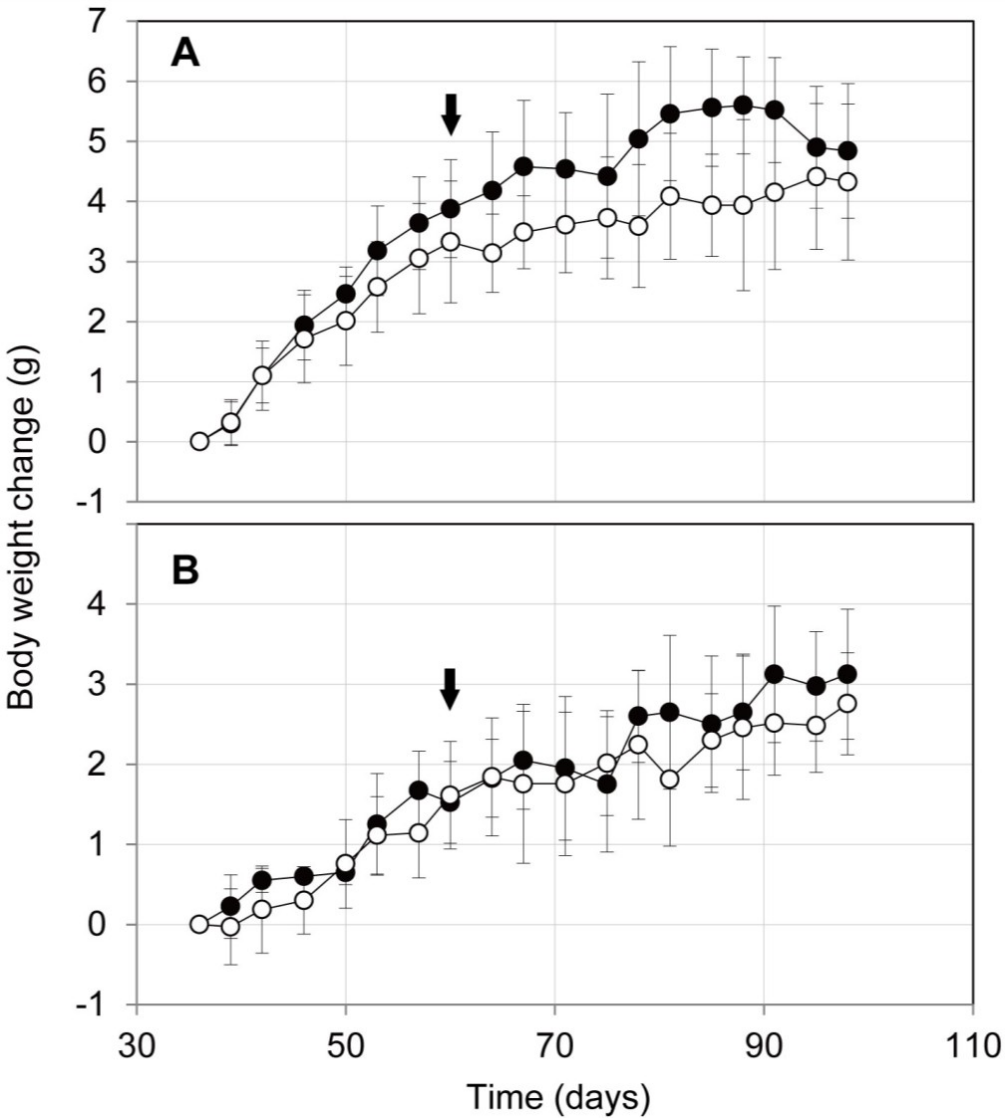
Body weight change of mouse with oral administrations of ProPS4. ProPS4 (10 mg dry weight/200 μL D.W.) was administered orally thrice weekly by a stomach tube to male (**A**) and female (**B**) C57BL/6J mice from 60 to 100 days old. Closed circles indicate a mean body weight change of mouse orally administered with ProPS4, and open circles indicate those without ProPS4. Arrows indicate the beginning of the oral administration. Error bars represent the standard deviation for the respective mean values.

To assess the influence of the oral administration of ProPS4 on mouse health, ProPS4 (10 mg dry weight/200 μL D.W.) was administered orally thrice weekly by a stomach tube to C57BL/6J mice for 40 days. The administration of 10 mg per mouse is comparable to 16 g per human with a body weight of 50 kg. Although ProPS4 was activated to PS4 in the digestive tract of the mouse as abovementioned, any serious health hazard was not observed in the C57BL/6J mice. The body weight change of the mouse during the administration is shown in Figure 2. The mean value of the change in the male mouse administered with ProPS4 was consistently higher than that of the control; however, there was no significant difference between the two groups. In the female mouse, there were few differences in the average of body weight change, and there was no significant difference between the administered group and control.

### 2.4. Influence of PS4 Injection on Kidney

**Figure 3:**
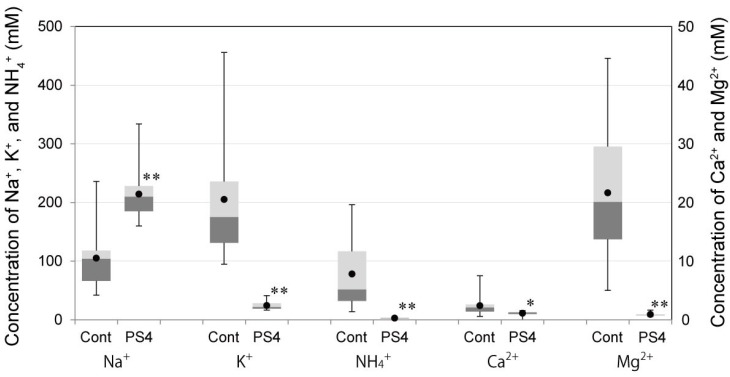
Cation concentrations in urine after administration of PS4. Three 7-week-old male ICR mice were injected with PS4 (10 μg in 150 μL PBS) by a subcutaneous route (PS4). Moreover, four mice were injected with 150 μL PBS as the control (Cont). The mice were then observed in a metabolic cage, and all urine was collected every hour until 24 h after administration. The concentrations of sodium, potassium, ammonium, calcium, and magnesium ions were determined by ion chromatography and are shown by a box plot. The mean values are indicated with closed circles. The value marked with the * symbol indicates that the value of the PS4-administered group is significantly different from the value of control groups (*: *p* < 0.05; **: *p* < 0.01). The number of sample was 16 (Cont) and 12 (PS4).

**Figure 4:**
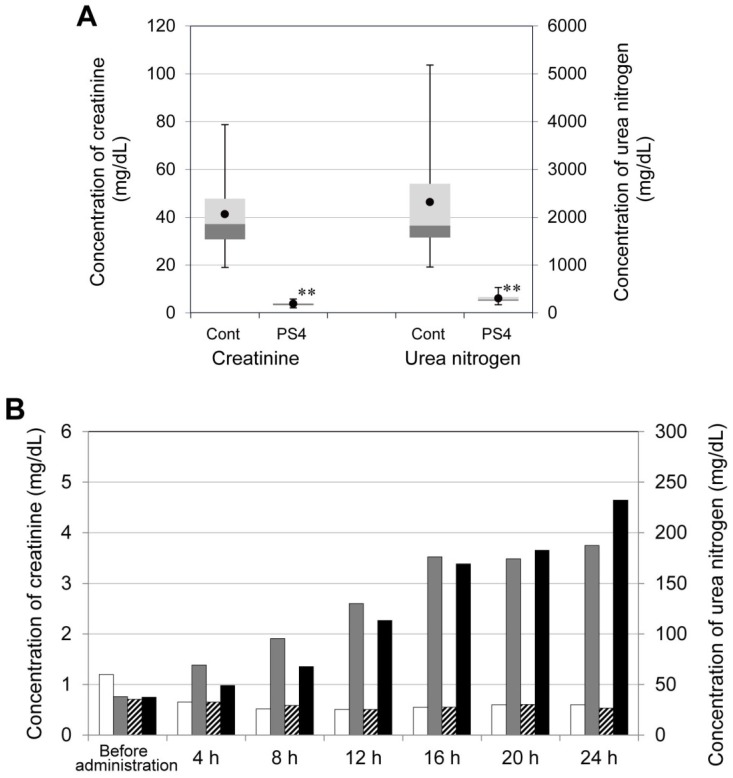
Concentrations of creatinine and urea nitrogen in urine and serum of mice administered with PS4. Male ICR mice were injected with 10 μg PS4 in 150 μL PBS (PS4) or 150 μL PBS (Cont) by a subcutaneous route. (**A**) A portion of them were observed in a metabolic cage, and their urine was collected every hour until 24 h after administration. The concentrations of creatinine and urea nitrogen in urine were determined and are shown by a box plot. Other conditions are the same as given in the legend of Figure 3. (**B**) One of the mice from the PS4-administered group and one from the control group were sacrificed every 4 h, and their blood samples were collected separately. The concentrations of creatinine and urea nitrogen in serum were determined. Gray bars indicate the concentration of creatinine in the serum of the mice administered with PS4, and white bars indicate that of the control group. Black bars indicate the concentration of urea nitrogen of the PS4-administered group, and hatched bars indicate that of the control group.

Male ICR mice were injected with 10 μg PS4 (in 150 μL PBS) by subcutaneous injection and subsequently observed in a metabolic cage. All of their urine was collected every hour until 24 h after administration. The urine of the PS4-administered mice was clear and colorless, although under control conditions was corn color. The concentrations of cations in the urine were determined and are shown in Figure 3. In the group of PS4-administered mice, the concentrations of potassium, ammonium, calcium, and magnesium ion were significantly low compared with those in the control group. Moreover, the concentration of sodium ion in the urine of administered group was significantly high compared with that of the control group (Figure 3). The differences in cation concentrations imply that kidney function was negatively affected by PS4 administration. Thus, creatinine and urea nitrogen in the urine and serum were determined and used as an index of the level of kidney function. In the urine, after PS4 injection, the concentrations of creatinine were less than 5.8 mg/dL, and those of urea nitrogen were less than 530 mg/dL. In the control group, concentrations of creatinine ranged from 19 to 79 mg/dL, and those of urea nitrogen ranged from 960 to 5200 mg/dL. Both concentrations in the administered group were significantly low compared with those in the control group (Figure 4A). In the serum, after the injection of PS4, both concentrations of creatinine and urea nitrogen increased over time, although those of the control group remained constant value during 24 h (Figure 4B). These results suggest that creatinine and urea nitrogen were not removed from serum by kidney and that kidney function was impaired by PS4 administration.

**Figure 5:**
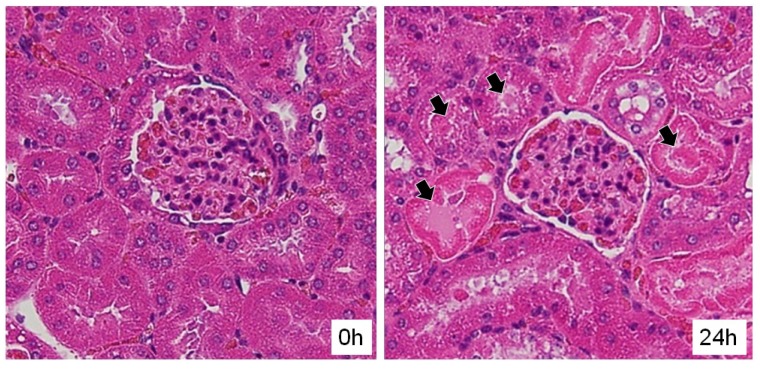
Slice of the mouse kidney before and after administration with PS4. The kidneys were fixed, sliced, and examined by hematoxylin-eosin staining. Black arrows indicate some materials in the proximal renal tubules.

Six 7-week-old male ICR mice were given an intraperitoneal injection of PS4 (20 μg in 200 μL PBS). Their kidneys were fixed, sliced, and examined by hematoxylin-eosin staining. Some cells of proximal renal tubules associated with necrosis and some materials in the tubules (Figure 5, black arrows) were observed in all the kidneys of the mice administered with PS4. These results suggest that the impairment of kidney function was caused by damage to the proximal renal tubule.

## 3. Experimental Section

### 3.1. Preparation of Inclusion Body of Recombinant ProPS4 and Purification of PS4

The inclusion body of recombinant ProPS4 was prepared by the same as the previously reported method [13] from *Escherichia coli* BL21 (DE3) cells transformed with a plasmid containing a gene for ProPS4. The pellet finally harvested was lyophilized and stored at 4 °C until use. PS4 was prepared from abovementioned ProPS4 inclusion body by the previously described purification method [9]. The purified PS4 was stored at −80 °C until use.

### 3.2. Animals

All animals were treated and handled in accordance with the national guidelines for care and use of laboratory animals and with the approval of the Fukuoka Industrial Technology Center Animal Care and Use Committee. Sixty-one ICR mice used in this study were purchased from Kyudo (Saga, Japan) in the age of 6 weeks. Two pairs of C57BL/6J mice were from Jackson Laboratory (Bar Harbor, ME, USA) in the age of 6 weeks, and then they were generated by breeding and used.

### 3.3. Acute Toxicity of PS4 in ICR Mice

PS4 was injected into 7-week-old male ICR mice by a subcutaneous route with 100 μL PBS. The dose amount and number of treated mice are shown in Table 1. The LD_50_ value and 95% confidence limit of the value were calculated by the probit analysis method [19].

### 3.4. Oral Administration of ProPS4 and Activation of the Toxin in the Digestive Tract

Dried inclusion body of ProPS4 was added to the powder of LabDiet 5085 (Labdiet, St. Louis, MO, USA) at a 1:9 weight ratio, and they were powdered in a mortar. The diet was provided *ad libitum* to four 20-week-old male C57BL/6J mice from 16:45 until 9:30 next morning. Subsequently, the mice were sacrificed, and the content in the digestive tract was collected. The small intestine was cut into quarters, and the content was collected separately. Contents in the large intestine were collected every fecaloma. The contents were measured (wet-weight) and suspended in a 10-fold volume of PBS containing 10 mM EDTA. The sample was disrupted for 30 s at 5000 rpm in the presence of 0.5 g of glass beads (φ1.0 mm) using a bead cell disrupter Micro Smash MS-100 (Tomy Seiko, Tokyo, Japan) two times. The sample was centrifuged at 5000 × *g* for 10 min, and 100 μL supernatant was collected. The solution was added to 100 μL PBS and then filtered using a centrifugal filter device (Amicon ultrafree-MC with 0.45 μm pore PVDF, Millipore, Bedford, MA, USA) for 3 min at 10,000 × *g*. The filtered sample was assayed for cytotoxic activity against CACO-2 cells using a previously described method [9]. Protein concentration of the sample was determined by the Bradford method [20]. Each sample containing 150 ng protein was analyzed by SDS-PAGE [21] with a polyacrylamide gradient gel (10%–20%; purchased from Wako Pure Chemical Industries, Osaka, Japan). Immunoblotting was performed with a rabbit antiserum raised against PS4 and commercially supplied HRP-conjugated anti-rabbit immunoglobulins (Dako, Glostrup, Denmark). Positive bands were detected with SuperSignal West Femto Maximum Sensitivity Substrate (Thermo Fisher Scientific, Waltham, MA, USA).

### 3.5. Body Weight Changes of Mice after Oral Administration of ProPS4

C57BL/6J mice were purchased from Jackson Laboratory. Then they were generated by breeding and used. The inclusion body of ProPS4 (10 mg dry weight/200 μL D.W.) was administered orally thrice weekly by a stomach tube into five male and four female mice. As the control, eight male and seven female mice were administered 200 μL D.W. in the same manner. The administration was continued from the age of 60 days to 100 days.

### 3.6. Concentrations of Cations, Creatinine, and Urea Nitrogen in Urine after Administration of PS4

Three male 7-week-old ICR mice were injected with 10 μg PS4 (in 150 μL PBS) by a subcutaneous route, and four mice were injected with 150 μL PBS as the control. They were observed in a metabolic cage, and all urine was collected every hour until 24 h after administration. Cation concentrations of urine samples (diluted 1000-fold) were determined by ion chromatography (Dionex DX-120, Thermo Fisher Scientific) with the use of super pure water containing 10 mM methanesulfonic acid as the eluent. Creatinine concentrations of urine samples (diluted 20-fold) were determined using a LabAssay Creatinine kit (Wako Pure Chemical Industries) based on an *in vitro* colorimetric Jaffé method [22] using a 96-well microplate. Urea nitrogen concentrations of urine samples (diluted 20-fold) were determined using a Urea Nitrogen kit UN3 (Wako Pure Chemical Industries) based on enzymatic determination with urease and glutamate dehydrogenase using a 96-well microplate [23]. The absorbance of the samples was measured with a microplate reader VersaMax (Molecular Devices, Sunnyvale, CA, USA).

### 3.7. Concentration of Creatinine and Urea Nitrogen in Mouse Serum after Administration of PS4

Seven 7-week-old male ICR mice were injected with 10 μg PS4 (in 150 μL PBS) and other 7 mice were injected with 150 μL PBS as the control. One of them was sacrificed every 4 h, and the blood was collected. All blood samples were preserved at 4 °C for a day, and the serum was collected by centrifugation at 1000 × *g* for 30 min. The concentrations of creatinine and urea nitrogen in serum were determined by the abovementioned method.

### 3.8. Preparation of Kidney Slices of Mice Administered with PS4

Eight 7-week-old male ICR mice were used. Six mice were given an intraperitoneal injection of PS4 (20 μg in 200 μL PBS). The kidneys of two mice were harvested 4, 8, and 24 h after injection. The kidneys of remaining two mice were also harvested as the control. The kidneys were fixed with 4% formaldehyde solution in PBS. Then they were embedded in paraffin, sectioned, and examined by hematoxylin-eosin staining.

## 4. Conclusions

The results obtained in this study indicate that ProPS4 can be solubilized and activated in the digestive tract of mouse and that its cytotoxic activity is retained in the stomach and small intestine. PS4 is an exceptional Bt toxin that is stable in acidic conditions. Although orally administered ProPS4 was solubilized and activated to PS4 in the digestive tract of C57BL/6J mice, any serious health hazard is not observed, and no significant difference in the body weight changes by comparing with the control is not observed. This finding is useful to explore the possibility for the treatment and prevention of gastrointestinal cancer by the oral administration of ProPS4. This study shows a subcutaneous injection of PS4 is lethal to mouse with the LD_50_ value of 0.16 mg/kg. Concentrations of cations, creatinine and urea nitrogen in the urine and serum indicate that PS4 impairs kidney function in mice. Moreover, slices of kidney with hematoxylin-eosin staining show that PS4 may induce damage of the proximal renal tubule of the kidney. The results obtained from the experiments may be valuable for further application of PS4 for cancer therapy.
